# S100‐A9 protein in exosomes derived from follicular fluid promotes inflammation via activation of NF‐κB pathway in polycystic ovary syndrome

**DOI:** 10.1111/jcmm.14642

**Published:** 2019-09-30

**Authors:** Han Li, Xin Huang, Xinwen Chang, Julei Yao, Qizhi He, Zhijun Shen, Yazhong Ji, Kai Wang

**Affiliations:** ^1^ Clinical and Translational Research Center Shanghai First Maternity and Infant Hospital Tongji University School of Medicine Shanghai China; ^2^ Department of Assisted Reproduction Shanghai First Maternity and Infant Hospital Tongji University School of Medicine Shanghai China; ^3^ Department of Pathology Shanghai First Maternity and Infant Hospital Tongji University School of Medicine Shanghai China; ^4^ Reproductive Medical Center Tongji Hospital Tongji University School of Medicine Shanghai China

**Keywords:** exosome, follicular fluid, polycystic ovary syndrome, protein profile/proteomic analysis

## Abstract

Exosomes have recently emerged as key mediators of different physiological and pathological processes. However, there has been few report about proteomic analysis of exosomes derived from human follicular fluid and their association with the occurrence of PCOS. Herein, we used TMT‐tagged quantitative proteomic approach to identify proteomic profiles in exosomes derived from follicular fluid of PCOS patients and healthy controls. We identified 662 proteins in exosomes derived from human ovarian follicular fluid. Eighty‐six differently expressed proteins (*P* < .05) were found between PCOS and healthy women. The alterations in the proteomic profile were related to the inflammation process, reactive oxygen species metabolic process, cell migration and proliferation. Importantly, we observed that follicular fluid exosomes contain S100 calcium‐binding protein A9 (S100‐A9) protein. Exosome‐enriched S100‐A9 significantly enhanced inflammation and disrupted steroidogenesis via activation of nuclear factor kappa B (NF‐κB) signalling pathway. These data demonstrate that exosomal proteins are differentially expressed in follicular fluid during disease process, and some proteins may play important roles in the regulation of granulosa cell function. These results highlight the importance of exosomes as extracellular communicators in ovarian follicular development.

## 1 INTRODUCTION

Polycystic ovary syndrome (PCOS) is a common endocrine disorder, with a prevalence around 5%‐10% in women.^1^, ^2^, ^3^, ^4^ PCOS is characterized by oligo‐ovulation or anovulation, hyperandrogenism and polycystic ovaries.^5^ In addition to reproductive abnormalities, PCOS is also associated with obesity,^6^ type 2 diabetes,^1^, ^7^ hyperinsulinaemia, insulin resistance,^8^, ^9^ lipid abnormalities and cardiovascular diseases.^10^, ^11^ However, the pathogenesis and molecular defects in PCOS are not fully understood.^12^

Follicular fluid (FF) provides an important microenvironment for follicular development and oocyte maturation. The accumulation of follicular fluid is mainly formed by the secretion of granulosa cells, theca cells and oocytes as well as by diffusion of plasma components from capillaries to the antrum.^13^, ^14^ The major components of follicular fluid are proteins, steroids, metabolites and polysaccharides.^15^, ^16^ Follicular fluid is the medium for bi‐directional communication between oocytes and the surrounding somatic cells.^17^ These cells can produce growth factors and cytokines that contribute to the regulation of ovarian function through paracrine/autocrine systems.^18^

Exosomes are small membrane‐enclosed vesicles (30‐100 nm in diameter) secreted by a wide range of living cells under normal or pathophysiological circumstances.^19^, ^20^ Exosomes contain several regulatory molecules, such as mRNAs, microRNAs (miRNAs), proteins and lipids.^21^ These compositions can be transferred between different types of cells and influence biological activities.^21^, ^22^, ^23^ Recently, some studies uncovered the potential function of exosomes derived from FF as carriers of miRNAs in steroidogenesis, follicular development and other pathological conditions.^24^, ^25^, ^26^, ^27^

Although proteome and peptidome were identificated in human FF,^28^, ^29^, ^30^, ^31^, ^32^, ^33^ little was known about the proteomic analysis of exosomes in follicular fluid and their potential roles in follicular development and reproduction‐related disorders. Therefore, we used an TMT‐tagged quantitative proteomic approach to compare the proteomic profile of exosomes derived from FF of PCOS and healthy women. Then, we focused on differently expressed proteins and several important biological pathways altered during PCOS progression. We found exosomes carrying S100 calcium‐binding protein A9 (S100‐A9) were able to activate the nuclear factor kappa B (NF‐κB) pathway, increase inflammation and disrupt steroidogenesis, which probably involved in the occurrence of PCOS.

## 2 MATERIALS AND METHODS

### 2.1 Study population and sample collection

All the participants included in the study were women undergoing IVF or ICSI at Shanghai First Maternity and Infant Hospital between February 2017 and December 2017. Patients for this study were divided into two groups: PCOS (n = 8) and matching control (n = 8). PCOS was defined according to the Rotterdam consensus criteria.^1^ The control group contained patients undergoing IVF due to male factor infertility or tubal factors. The exclusion criteria for both groups included women with endometriosis, cancer, premature ovarian insufficiency (POI) or other medical disorders that could affect follicular development. The information of involved patients is shown in Table 1. This study was approved by the Scientific and Ethical Committee of the Shanghai First Maternity and Infant Hospital affiliated to Tongji University. All patients underwent controlled ovarian hyperstimulation using a combination of GnRH agonist and recombinant FSH. Follicular fluid (2‐3 mL) was collected from the dominant follicles (>18 mm) by transvaginal ultrasound guided aspiration, 34‐36 hours after 10 000 IU human chorionic gonadotropin (hCG) administration. The samples were centrifuged at 3000 *g* for 15 minutes to remove cells debris and other particles. Supernatants were stored at −80°C for further use.

**Table 1:** Clinical characteristics of PCOS and normal patients

Parameters	Control (n = 8)	PCOS (n = 8)	*P*
Age (y)	30.50 ± 1.21	28.60 ± 1.19	.28
BMI (kg/m^2^)	21.71 ± 0.61	21.11 ± 0.80	.56
Basal serum FSH (mIU/mL)	6.08 ± 0.43	5.56 ± 0.56	.46
Basal serum LH (mIU/mL)	3.20 ± 0.49	9.37 ± 3.01	.06
Basal serum T (ng/mL)	0.38 ± 0.04	0.66 ± 0.07	.003
Basal serum oestradiol (pg/mL)	44.81 ± 6.32	106.2 ± 45.02	.19
Total FSH administration (IU)	1664 ± 238.7	1489 ± 145.4	.54
Number of oocytes retrieved	11.80 ± 1.65	14.10 ± 2.64	.47

Note

All results are presented as the mean ± SEM.

Abbreviations: BMI, body mass index; FSH, follicle‐stimulating hormone; LH, luteinizing hormone; T, testosterone.

### 2.2 Exosome isolation

The follicular fluid exosomes were collected by ExoQuick‐TC Exosome Precipitation Solution Kit (System Biosciences). In brief, 250 µL ExoQuick Solution was added to the 1 mL follicular fluid (1:4), mixed well and incubated overnight at 4°C. Then, the mixture was centrifuged at 1500 *g* for 30 minutes at 4°C, and the supernatant was removed. The exosome pellet was resuspended in 500 µL PBS and passed through a 0.22‐µm filter.

Exosomes from cell culture supernatants were isolated by ultracentrifugation. In brief, 293T cells were starved in medium containing 1% BSA for 48 hours. Then, cell supernatant was centrifuged at 300 *g* for 10 minutes to remove cells. The supernatant fluid was then centrifuged at 2000 *g* for 10 minutes at 4°C to remove dead cells. The resultant supernatant fluid was transferred to an ultracentrifuge tube and centrifuged at 100 000 *g* for 2 hours. The pellet was suspended in PBS and filtered through a 0.22‐μm filter, and then centrifuged at 100 000 *g* for 2 hours. The pellet was resuspended in 200 μL PBS and stored at −80°C.

### 2.3 Transmission electron microscopy

Exosomes were analysed by transmission electron microscopy (TEM) as previously described.^34^, ^35^ A total of 20 μL of exosome suspension (5 µg/µL) was fixed on a continuous grid and then negatively stained with 2% uranyl acetate solution for 1 minute and air‐dried. The samples were observed by FEI Tecnai G2 spirit transmission electron microscope (FEITM) at an acceleration voltage of 120 kV.

### 2.4 Nanoparticle tracking analysis

Nanoparticle tracking analysis (NTA) measurements were performed using a NanoSight NS300 instrument (Malvern Panalytical) with a 488‐nm laser and sCMOS camera module (Malvern Panalytical). Measurements in flow mode were performed with a flow rate of 50, these flow measurements consisted of 3 measurements of 60 seconds, and the captured data were analysed using NTA 3.2 software.

### 2.5 Proteomic analysis

Protein concentration in the supernatant was determined with BCA kit (Beyotime) according to the manufacturer's instructions. After trypsin digestion, peptide was desalted by Strata X C18 SPE column (Phenomenex) and vacuum‐dried. Peptide was reconstituted in 0.5 M TEAB and processed according to the manufacturer's protocol for TMT kit. The tryptic peptides were fractionated into fractions by high pH reverse‐phase HPLC using Agilent 300Extend C18 column (5 μm particles, 4.6 mm ID, 250 mm length). For LC‐MS/MS analysis, the tryptic peptides were dissolved in 0.1% formic acid (solvent A), directly loaded onto a home‐made reversed‐phase analytical column (15‐cm length, 75 μm i.d.). The gradient was comprised of an increase from 6% to 23% solvent B (0.1% formic acid in 98% acetonitrile) over 26 minutes, 23%‐35% in 8 minutes and climbing to 80% in 3 minutes then holding at 80% for the last 3 minutes, all at a constant flow rate of 400 nL/min on an EASY‐nLC 1000 UPLC system. The peptides were subjected to NSI source followed by tandem mass spectrometry (MS/MS) in Q Exactive™ Plus (Thermo) coupled online to the UPLC. The resulting MS/MS data were processed using MaxQuant search engine (v.1.5.2.8). The gene ontology analysis was derived from the UniProt‐GOA database (http://www.http://www.ebi.ac.uk/GOA/) and GO annotation (http://geneontology.org/). The Kyoto Encyclopedia of Genes and Genomes (KEGG) connects known information on molecular interaction networks.

### 2.6 Western blot analysis

The concentration of proteins was quantified using the Pierce BCA Protein Assay Kit (Thermo Fisher Scientific) following the manufacturer's instructions. Proteins were separated by 12% SDS‐PAGE gels and transferred to PVDF membranes by gel electrophoresis and electroblotting, respectively. After blocking with 5% BSA, blots were probed with primary antibodies at 4°C overnight. Then, membranes were washed and incubated with second antibodies. Ultimately, proteins were visualized using the enhanced chemiluminescence reagents (Thermo Fisher Scientific). The antibodies we used are listed in Table S2. The relative protein expression levels were analysed by densitometry using the ImageJ imaging analysis software (NIH).

### 2.7 ELISA

Concentration of S100‐A9 protein in 100 μL follicular fluid, as well as in exosomes derived from 100 μL follicular fluid (FF‐Exos), was measured by commercial ELISA kits (Boster Biological Technology) according to the manufacturer's instruction. In order to prepare exosome lysis solution, 25 µL ExoQuick Exosome Precipitation Solution was added to the 100 μL follicular fluid, mixed well and incubated overnight at 4°C. Then, the mixture was centrifuged at 1500 *g* for 30 minutes at 4°C. The exosome pellet was resuspended in 100 µL PBS, repeated freeze‐thaw cycles three times to gain exosome lysis solution.

### 2.8 Construction of S100‐A9‐enriched exosomes

The expression vector S100‐A9 (pBABE‐puro‐S100‐A9) and its control vector (pBABE‐puro) were constructed and transfected into the Platinum‐E packaging cell line using Lipofectamine TM 2000 (Invitrogen). Forty‐eight h after transfection, supernatants were harvested and used to infect the packaging cell line 293T for 48 hours in the presence of 4 ng/mL polybrene. After selection by puromycin and culture expansion, the stable cell clones overexpressing S100‐A9 were attained. And the infected cells with an empty pBABE‐puro vector were used as control. Quantitative RT‐PCR was used to detected S100‐A9 expression level in constructed 293T cells. Western blot was used to analyse the S100‐A9 protein level in constructed 293T cells and their exosomes.

### 2.9 Cell culture

A steroidogenic human granulosa‐like tumour cell line (KGN) was donated by Dr Yugui Cui of the State Key Laboratory of Reproductive Medicine, The First Affiliated Hospital of Nanjing Medical University of China. The cells were frozen in serum‐free cell freezing medium (HAKATA, Chuan Qiu Biotechnology). The cells were cultured in DMEM/F12 medium (Sigma‐Aldrich) supplemented with 10% foetal bovine serum, 1% penicillin/streptomycin at 37°C, 5% CO_2_. To explore the potential effects of S100‐A9 protein in exosomes, KGN cells were incubated with 10 µg/mL S100A9 protein (Sino Biological) or 100 µg/mL exosomes secreted by 293T cell which enriched S100‐A9. To inhibit the NF‐κB pathway, the cells were pre‐treated for 1 hour with BAY‐117082 (NF‐κB inhibitor, 10 µM; Beyotime).

### 2.10 Exosome uptake assay

For exosome uptake analysis, exosomes isolated from engineered 293T cells were labelled with the red CM‐Dil membrane dye (Thermo Fisher Scientific) and incubated 5 minutes at 37°C. The working concentration of CM‐Dil was 1 mM. A mixture without exosomes was used as a negative control. The labelled exosomes were collected by ExoQuick‐TC Kit according to the manufacturer's instructions.

Approximately 2000 KGN cells were cultured in a confocal cell culture dish. The cells were starved for 12 hours and then labelled with a green fluorescent dye PKH67 (MINI67‐1KT, Sigma‐Aldrich) according to the manufacturer's protocol. Then, the cells were washed twice with PBS and incubated with labelled exosomes (100 µg/mL) for 24 hours at 37°C, 5% CO_2_. After incubation, the cells were fixed with 4% paraformaldehyde for 20 minutes at room temperature. Nuclei were stained with DAPI. The signals were examined by confocal microscopy (TCS SP8; Leica).

### 2.11 Cell proliferation assay

To assess the effect of exosomal protein S100‐A9 on cell proliferation, about 3000 KGN cells/well were seeded in a 96‐well plate with complete DMEM/F12. After 24‐hour attachment, the cells were starved for 12 hours and then treated with different dose of exosomes which overexpressed S100‐A9. After 48‐hour incubation, cell proliferation was determined using the CellTiter 96 AQueous One Solution Cell Proliferation Assay Kit (Promega) according to the manufacturer's instructions.

### 2.12 RNA isolation and quantitative RT‐PCR

Total RNA was extracted using TRIzol (Invitrogen), and RNA was then reverse‐transcribed using SuperScript First‐Strand cDNA System (Takara) according to the manufacturer's instructions. Quantitative RT‐PCR (qRT‐PCR) was performed using the SYBR Green PCR master mix (Takara) and the StepOnePlus PCR system (Thermo Fisher Scientific) according to the manufacturer's instructions. The housekeeping gene GAPDH was used as an endogenous control. The primer sequences are shown in Table S1.

### 2.13 Luminex array assay of cytokine expression

KGN cells were seeded in 6‐well plates at 1 × 10^5^ per well, and after 24‐hour attachment, the cells were starved for 12 hours and then treated with 100 µg/mL exo‐S100‐A9. After 24‐hour incubation, the culture medium was centrifuged at 3000 *g* for 5 minutes. The concentrations of cytokines in the cell culture supernatants were assayed using a human 27‐Plex cytokine antibody array to examine IL‐1 β, IL‐1ra, IL‐2, IL‐4, IL‐5, IL‐6, IL‐7, IL‐8, IL‐9, IL‐10, IL‐12 (p70), IL‐13, IL‐15, IL‐17, basic FGF, eotaxin, G‐CSF, GM‐CSF, IFN‐γ, IP‐10, MCP‐1 (MCAF), MIP‐1α, MIP‐1β, PDGF‐BB, RANTES, TNF‐α and VEGF‐A according to the manufacturer's protocol.

### 2.14 Statistical analyses

Data were expressed as the mean ± standard error of the mean (SEM) of at least three independent times. All statistics were performed with SigmaStat, v.3.5 (Jandel Co.). Statistical significance was analysed by unpaired Student's *t* tests or one‐way ANOVA. A *P*‐value < .05 was considered statistically significant.

## 3 RESULTS

### 3.1 Isolation and identification of exosomes

**Figure 1:**
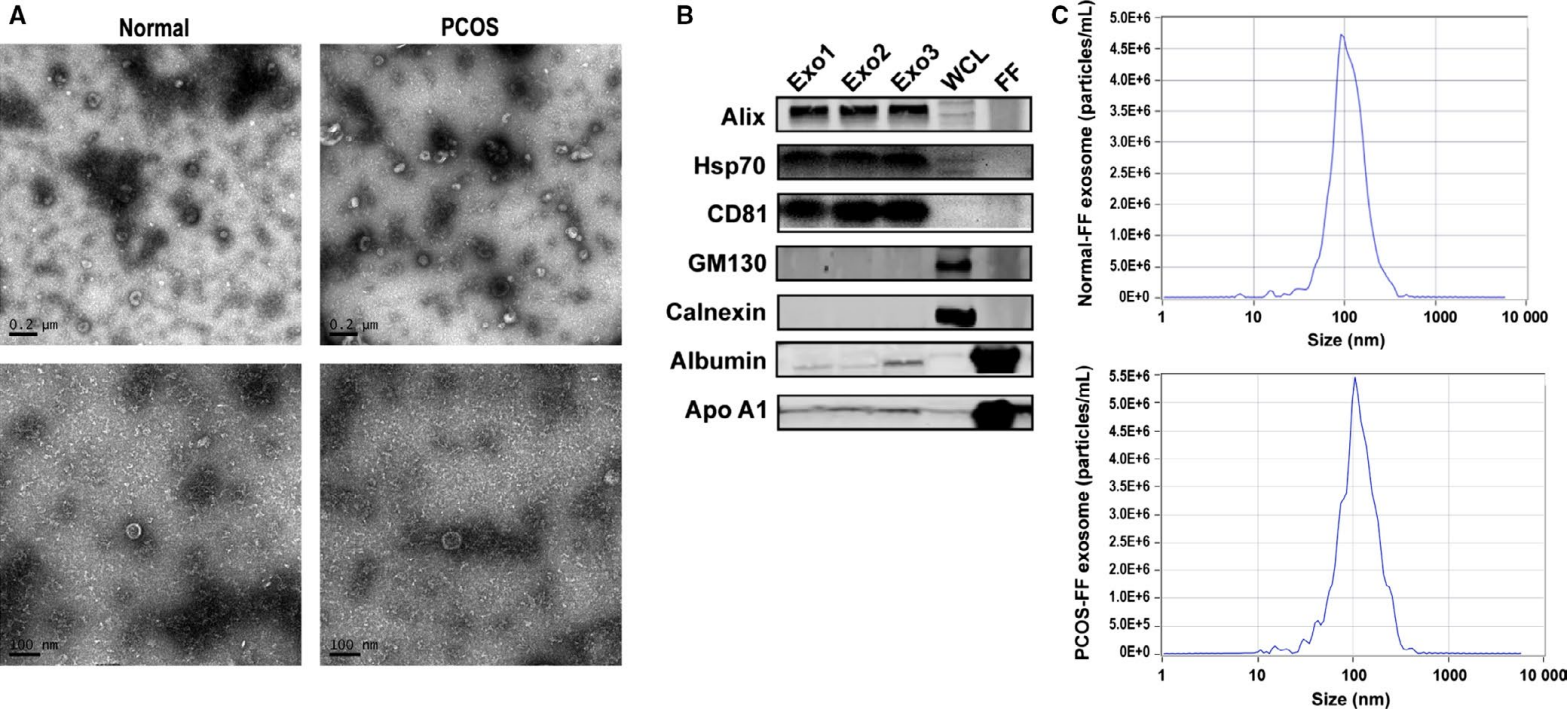
Characterization of exosomes derived from human follicular fluid. A, Transmission electron micrographs (TEM) of exosomes derived from follicular fluid of normal control and PCOS women. Scale bar, 200 nm and 100 nm. B, The positive exosomal marker (Alix, Hsp70, CD81), negative marker (Calnexin and MG130), purity control (apolipoprotein A1 and albumin) immunoblots of follicular fluid exosomes (exo1‐3), whole cell lysis of granulosa cells (WCL) and follicular fluid (FF). C, The representative nanoparticle tracking analysis (NTA) profile of exosomes from human follicular fluid of normal (normal‐FF) and PCOS (PCOS‐FF)

Exosomes derived from follicular fluid were characterized by Western blot, TEM and NTA. The positive exosomal marker (Alix, Hsp70, CD81), negative marker (Calnexin and MG130), purity control (apolipoprotein A1 and albumin) immunoblots of follicular fluid exosomes, whole cell lysis of granulosa cells (WCL) and follicular fluid (FF) are shown in Figure 1B. NTA and TEM results (Figure 1A,C) were consistent with previously reported characteristics of exosomes.^26^, ^36^ TEM image of microvesicles (MVs) from FF (particles >100 nm) as negative control is shown in Figure S2A.

### 3.2 Proteomic analysis of differentially expressed exosomal proteins

**Figure 2:**
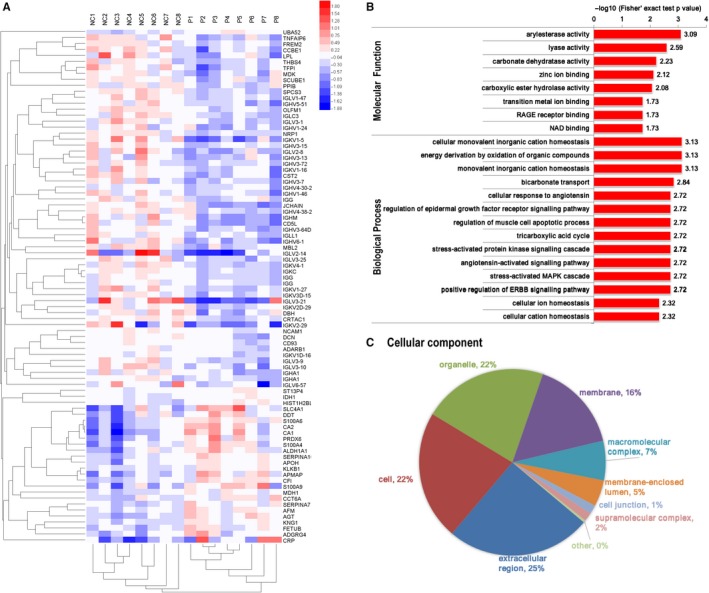
Proteomic analysis of follicular fluid exosomes derived from normal and PCOS follicular fluid. A, Heat map of differentially expressed exosomal proteins from PCOS (n = 8) and normal control (n = 8). B, Molecular function and biological process analysis for differentially expressed exosomal proteins derived from follicular fluid of PCOS patients and health control. C, Cellular component analysis for differentially expressed exosomal proteins derived from follicular fluid of PCOS patients and health control

Proteomic analysis using TMT technology was used to detect the proteomic profiles in exosomes from normal and PCOS patients. The proteins which were detectable in at least one samples were included as quantifiable proteins in the analysis. In total, 662 proteins were identified, among which 546 proteins were quantified in control group, 547 proteins were quantified in PCOS. The quantifiable protein expression profiles of two groups were essentially the same. Differentially expressed proteins were identified with a cut‐off of absolute fold change ≥1.2 and *P*‐value < .05. The results showed that 86 proteins were differentially expressed between PCOS and normal control (Figure 2A). Among them, 27 exosomal proteins were significantly higher and 59 proteins were lower in PCOS patients than controls. Most differentially expressed proteins are listed in Table 2.

**Table 2:** Significantly differently expressed proteins in FF‐exosomes of PCOS and normal controls

Protein accession	Protein name	Fold of change (PCOS/Ctrl)	*P*‐value
Q8IZF6	Adhesion G‐protein‐coupled receptor G4	1.79	.0237
P02730	Band 3 anion transport protein	1.76	.0183
P00918	Carbonic anhydrase 2	1.58	.0130
P06702	Protein S100‐A9	1.56	.0256
P06703	Protein S100‐A6	1.55	.0048
Q9HDC9	Adipocyte plasma membrane‐associated protein	1.48	.0244
P30041	Peroxiredoxin‐6	1.46	.0257
P30046	d‐Dopachrome decarboxylase	1.46	.0166
P26447	Protein S100‐A4	1.46	.0151
P01019	Angiotensinogen	1.45	.0010
P00352	Retinal dehydrogenase 1	1.39	.0212
P05156	Complement factor I	1.32	.0013
Q9UGM5	Fetuin‐B	1.26	.0276
P05543	Thyroxine‐binding globulin	1.25	.0301
Q9UK55	Protein Z‐dependent protease inhibitor	1.25	.0219
P43652	Afamin	1.25	.0142
O75874	Isocitrate dehydrogenase [NADP] cytoplasmic	1.24	.0184
P02749	Beta‐2‐glycoprotein 1	1.23	.0465
P62987	Ubiquitin‐60S ribosomal protein L40	1.22	.0016
P01042	Kininogen‐1	1.21	.0089
P03952	Plasma kallikrein 1	1.20	.0068
P40925	Malate dehydrogenase, cytoplasmic	1.20	.0204
P09228	Cystatin‐SA	0.52	.0003
P10646	Tissue factor pathway inhibitor	0.66	.0049
P35443	Thrombospondin‐4	0.80	.0435
Q8IWY4	Signal peptide, CUB and EGF‐like domain‐containing protein 1	0.82	.0441
P61009	Signal peptidase complex subunit 3	0.70	.0001
P23284	Peptidyl‐prolyl cis‐trans isomerase B	0.80	.0354
Q99784	Noelin	0.76	.0018
O14786	Neuropilin‐1	0.76	.0009
P13591	Neural cell adhesion molecule 1	0.77	.0119
P21741	Midkine	0.76	.0269
O43866	CD5 antigen‐like	0.62	.0007
P07585	Decorin	0.64	.0005
Q6UXH8	Collagen and calcium‐binding EGF domain‐containing protein 1	0.65	.0020
P06858	Lipoprotein lipase	0.65	.0435
P10646	Tissue factor pathway inhibitor	0.66	.0049
P01721	Immunoglobulin lambda variable 6‐57	0.67	.0471
P98066	Tumour necrosis factor‐inducible gene 6 protein	0.68	.0048
Q9NPY3	Complement component C1q receptor	0.71	.0092

To select the proteins involved in PCOS disease progression, we focused on the proteins that differentially expressed between two groups. KEGG pathway and GO analysis were conducted to classify the affected biological functions, including cell component, biological process and molecular function (Figure 2B,C). The up‐regulated proteins in PCOS were involved in regulation of multiple biological processes and metabolism, such as stress‐activated protein signalling cascade, angiotensin‐activated signalling pathway, ERBB signalling pathway, MAPK cascade and epidermal growth factor receptor signalling pathway. We found several differentially regulated proteins in exosomes are related to regulation of inflammatory response and reactive oxygen species metabolic process, such as C‐reactive protein (CRP), S100‐A4, S100‐A9, peroxiredoxin‐6 (PRDX6), d‐dopachrome decarboxylase (DDT), retinal dehydrogenase 1 (ALDH1A1), angiotensinogen and kininogen‐1 (KNG1). Several proteins involved in the regulation of extrinsic apoptotic signalling pathway were down‐regulated, such as neuropilin‐1 and dopamine beta‐hydroxylase (DBH). Subcellular location analysis of differentially expressed exosomal proteins in follicular fluid of women with PCOS showed about 68% of these proteins are extracellular in origin (Figure S1A). The respective analysis about subcellular location of up‐ or down‐regulated exosomal proteins in PCOS is shown in Figure S1B,C. Cellular component of these proteins (Figure 2C) showed proteins of extracellular region, organelle, membrane and intracellular proteins were altered in PCOS.

**Figure 3:**
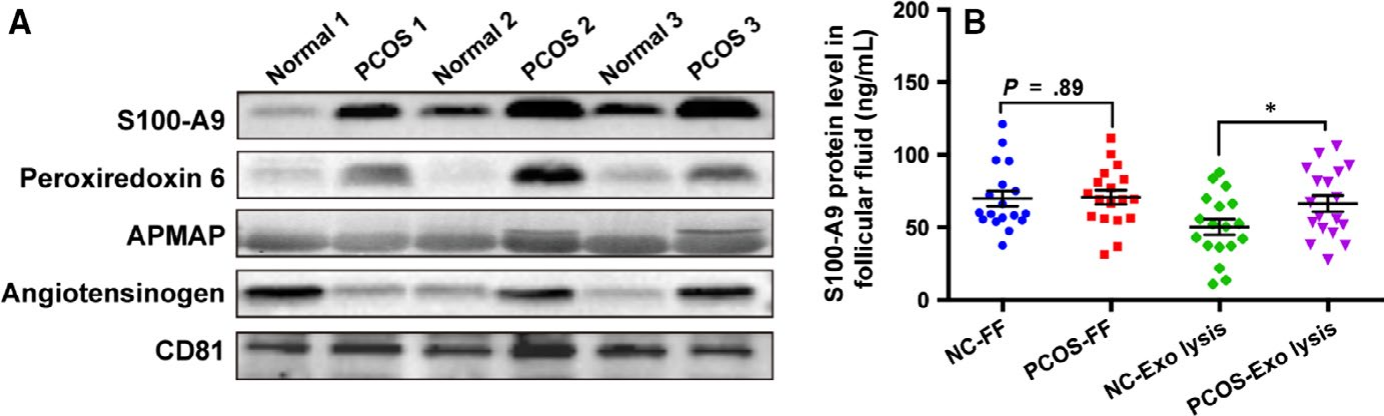
Validation of expression of several differentially expressed exosomal proteins. A, The representative image of expression of up‐regulated exosomal proteins S100‐A9, peroxiredoxin 6, APMAP and angiotensinogen by Western blot in exosomes from normal and PCOS follicular fluid (n = 6 per group). B, Concentration of S100‐A9 in follicular fluid supernatant (100 µL) and exosome lysis solution (exosomes were isolated from 100 µL FF, repeated freeze‐thaw cycles three times to gain lysis solution) measured by ELISA (n = 18 per group), *P < .05. All data were presented as means ± SEM. Exo‐lysis, exosome lysis solution; FF, follicular fluid; NC, normal control

Among the up‐regulated proteins detected in PCOS, we chose four proteins (angiotensinogen, peroxiredoxin‐6, S100‐A9 and APMAP), validated the expression of these proteins by Western blot. The protein selection criteria include relatively high expression level; a cut‐off of absolute fold change ≥1.4 between two groups; GO analysis (UniProt website); or published literatures indicate that this protein might be associated with PCOS pathological processes. Our results confirmed the presence of these proteins in exosomes derived from follicular fluid (FF‐Exos) of PCOS patients, whereas low presence was detected in healthy controls (Figure 3A).

### 3.3 S100‐A9 expression level in follicular fluid and FF‐Exos

S100‐A9 is an activator of the NF‐κB pathway, and its function is associated with inflammation.^37^ We measured the expression level of S100‐A9 in follicular fluid and FF‐Exos lysis solution by ELISA. As shown in Figure 3B, concentration of S100‐A9 was significantly higher in FF‐Exos of PCOS patients compared with controls, whereas no significant difference was found in the supernatant of follicular fluid between PCOS and control group.

### 3.4 Expression levels of S100‐A9 receptors

S100‐A9 can bind to the receptor of advanced glycation end products (RAGE),^38^ Toll‐like receptor 4 (TLR4)^38^ as well as extracellular matrix metalloproteinase inducer (EMMPRIN, CD147),^39^ which might play roles in mediating inflammatory effects of S100‐A9. Our western blot results showed S100‐A9 receptors (TLR4, EMMPRIN, RAGE) were expressed in granulosa cells of normal control and PCOS women and KGN cell line (Figure S2B).

### 3.5 Construction of S100‐A9‐enriched exosomes

**Figure 4:**
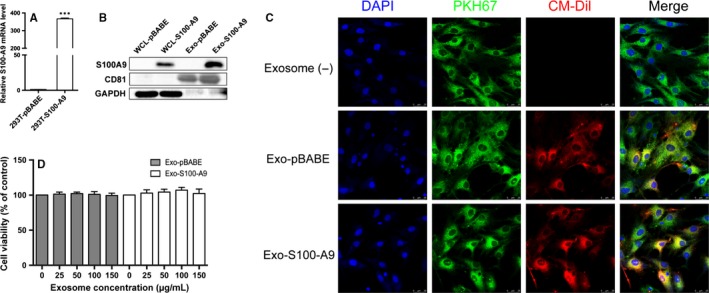
Construction of S100‐A9‐enriched exosomes. A, S100‐A9 mRNA expression level in 293T cells which stably transfected with empty pBABE‐puro vector (named 293T‐pBABE) or pBABE‐puro‐S100‐A9 vector (named 293T‐S100‐A9). ***P < .001. B, Western blot analysis of S100‐A9 expression level in 293T whole cell lysate (WCL) and exosomes from 293T cells culture supernatants. C, The detection of constructed exosome uptake by KGNs in vitro. KGNs that had been incubated for 24 h with CM‐Dil labelled exosomes are shown (CM‐Dil in red, PKH67 in green, DAPI in blue). Scale bar, 25 µm. D, Cell viability when treated with varying concentrations of control or S100‐A9‐enriched exosomes (0, 25, 50, 100 and 150 µg/mL) for 48 h. All values were presented as the means ± SEM of three pairs of independent experiments performed

293T is a useful cellular model.^34^ Herein, we generated 293T cell line overexpressing S100‐A9, which could produce S100‐A9 protein‐enriched exosomes. S100‐A9 mRNA expression level in 293T cells transfected with pBABE‐puro‐S100‐A9 vector was significantly higher than 293T cells transfected with empty vector (Figure 4A). Western blot analysis showed S100‐A9 protein expression level was significantly increased in 293T whole cell lysate and 293T cell‐secreted exosomes (Figure 4B). Since appropriate function of granulosa cells is critical for normal follicular development, we selected the most widely used granulosa‐like tumour cell lines, KGN cells, to explore the effect of S100‐A9 on its functions. The exosome uptake experiment confirmed this kind of exosome could be uptook by KGNs (Figure 4C). Cell viability assay indicated that control exosome (exo‐pBABE) and S100‐A9‐enriched exosome (exo‐S100‐A9) did not significantly impact cell viability (Figure 4D).

### 3.6 Exosomal S100‐A9 promotes NF‐κB pathway activation

**Figure 5:**
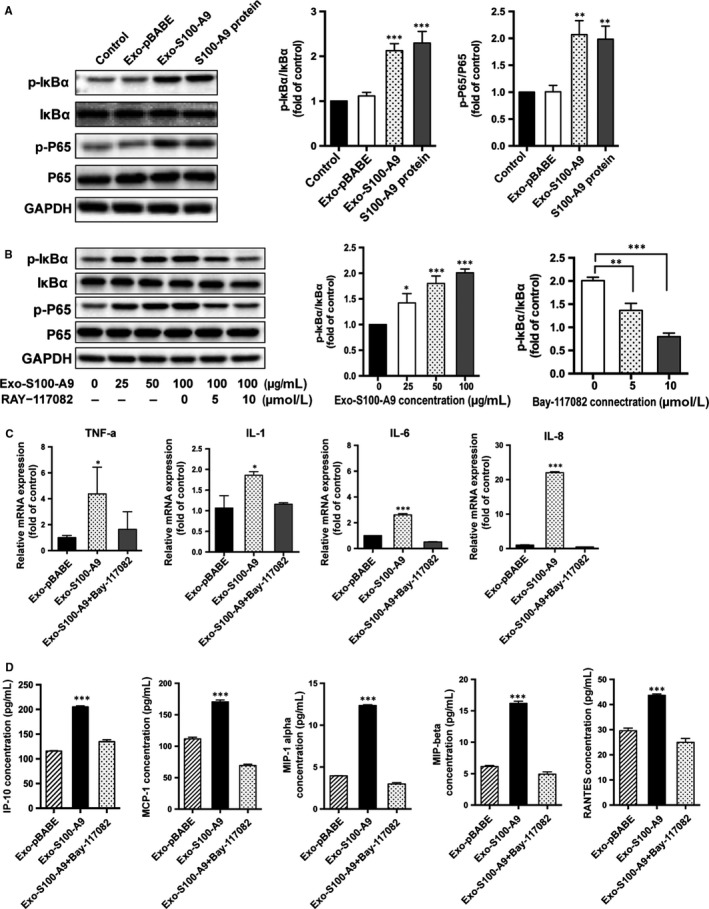
Exosomes overexpressed S100‐A9 promote NF‐κB pathway activation and inflammation in KGNs. A, Western blot analysis of NF‐κB pathway after in vitro incubation with exosomes overexpressed S100‐A9 (Exo‐S100‐A9) and exosomes from 293T cells which transfected with empty vector (Exo‐pBABE) (30 min; 100 µg/mL). PBS was added in control group. S100‐A9 protein was used as a positive control. B, Western blot analysis of NF‐κB pathway after KGN cells were treated with varying concentrations of S100‐A9‐enriched exosomes (0, 25, 50 or 100 µg/mL; 30 min) or NF‐κB pathway inhibitor BAY‐117082 (5, 10 µM; pre‐treated for 1 h, then treated with 100 µg/mL exosomes for 30 min). C, Relative mRNA expressions of inflammatory factors TNF‐α, IL‐1, IL‐6 and IL‐8 in KGNs. D, Concentrations of several chemokines in the KGNs culture supernatants after S100‐A9‐enriched exosome treatments (100 µg/mL, 24 h) or NF‐κB pathway inhibitor treatments (10 µM, pre‐treated for 1 h, then treated with 100 µg/mL exosomes for 24 h). *P < .05, **P < .01, ***P < .001 for comparison with exo‐pBABE. All values were the means ± SEM

S100‐A9 plays a role in inflammation process and is associated with the pathogenesis of various diseases.^37^, ^40^ We examined the activation of the NF‐κB in KGNs after incubated with 100 µg/mL exo‐S100‐A9. As shown in Figure 5A, IκB‐α and p65 phosphorylation were increased significantly after incubation with the S100‐A9‐enriched exosomes, indicating exosomal S100‐A9 could promote NF‐κB pathway activation in KGN cells. This activation is in a S100‐A9 dose‐dependent manner (Figure 5B). Moreover, exo‐S100‐A9‐induced NF**‐**κB activation could be suppressed by NF‐κB pathway specific inhibitor BAY‐117082 (Figure 5B). These results confirmed exosomal S100‐A9 was able to activate NF‐κB pathway in KGNs.

### 3.7 S100‐A9 enhances inflammation in KGN cells

**Figure 6:**
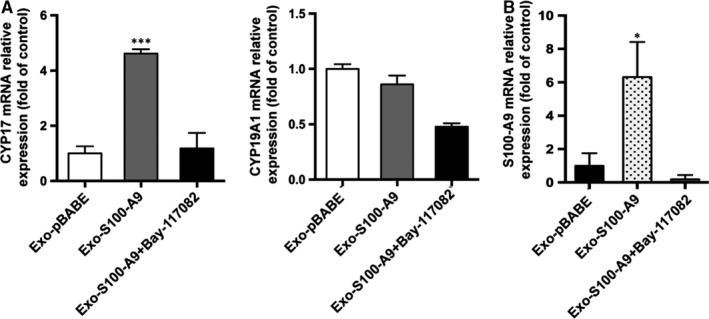
Exosomes overexpressed S100‐A9 disturb the expression of steroidogenesis‐related genes in KGNs. A, CYP17 and CYP19A1 mRNA expression and B, S100‐A9 mRNA expression level in KGNs after exosomes (100 µg/mL, 24 h) or NF‐κB pathway inhibitor treatments (10 µM, pre‐treated for 1 h, then treated with 100 µg/mL exosomes for 24 h). *P < .05, **P < .01, ***P < .001 for comparison with exo‐pBABE. All values were presented as the means ± SEM of three independent experiments performed

It is well known that NF‐κB is a key regulator which involved in the initiation of many pro‐inflammatory cytokines and enzymes. We found the level of TNF‐α, IL‐1, IL‐6 and IL‐8 mRNA in KGNs was significantly increased after exo‐S100‐A9 stimulation (Figure 5C). Among them, IL‐8 increased almost 20‐fold (Figure 6A). The concentrations of several chemokines in culture supernatants, such as IP‐10, MCP‐1, MIP‐1 alpha, MIP‐1 beta and RANTES, were all up‐regulated after exo‐S100‐A9 treatment (Figure 5D). The concentrations of G‐CSF, IL‐8 and IL‐6 in cell culture supernatants were also 2–3‐fold higher in exo‐S100‐A9‐treated group than control (Figure S1D). These inflammatory responses were partly or completely suppressed when KGN cells were pre‐treated with NF‐κB pathway inhibitor.

### 3.8 S100‐A9 affects expression of genes associated with steroidogenesis

CYP19A1 and CYP17 are key enzymes in androgen metabolic pathways. Increased activity of CYP17 has been hypothesized to enhance androgen biosynthesis and secretion in PCOS.^41^ To investigate the role of S100‐A9 in steroidogenesis, the expression levels of CYP17 and CYP19A1 mRNA in KGNs were determined by qRT‐PCR. We found that S100‐A9 significantly increased the CYP17 mRNA expression, whereas slightly suppressed CYP19A1 expression (Figure 6A). We also found AMHR, AR, ESR1 and ESR2 expression levels were dysregulated after exo‐S100‐A9 treatment (Figure S1E). The effect of S100‐A9 on expression of these genes was suppressed by NF‐κB pathway inhibitor. These results suggested S100‐A9 disturbed several genes expression and hormone signalling mainly through a NF‐κB pathway‐dependent manner.

## 4 DISCUSSION

Microenvironment signals in follicles are provided not only by gap junctions, paracrine, autocrine or endocrine signalling factors, but also by exosomes, which constitute a new communication mechanism.^42^ The miRNAs, lipids and proteins contained in exosomes can alter several essential biological processes and contribute to the progression of reproductive and obstetric related diseases.^43^ Previous study identified miRNAs in exosomes derived from human follicular fluid play important roles in steroidogenesis and closely associated with PCOS.^24^ However, no studies have investigated the proteomic profile of exosomes derived from human ovarian follicular fluid in PCOS patients so far.

We hereby reported that healthy and PCOS women follicular fluid‐derived exosomes represent different proteomic profiles. To our knowledge, this is the first time to identify the protein profile in the exosomes derived from women follicular fluid. Proteomic analysis of 86 proteins was differentially expressed between PCOS and normal control. When comparing our data with previous studies about differently expressed proteins associated with PCOS in follicular fluid or granulosa cells,^44^, ^45^, ^46^ only a few proteins were overlapped, such as afamin and kininogen. These results suggested exosomes have special contents and functions in biological and pathological conditions. Particularly, our results showed several differentially regulated proteins in exosomes play important roles in the regulation of inflammatory response and reactive oxygen species metabolic process. Consistent with our findings, several studies have suggested that PCOS is an inflammatory condition with increased levels of inflammatory markers in follicular fluid and plasma, as well as increased macrophages/mononuclear cell recruitment and local inflammation in the ovary.^47^, ^48^

To identify the role of exosomal proteins during PCOS progression, we focused on the S100‐A9 protein, which was up‐regulated in exosomes of PCOS patients. S100‐A9 belongs to a family of 25 homologous low‐molecular‐weight intracellular calcium‐binding proteins.^49^ It is well known that S100‐A8/S100‐A9 are mainly released by activated granulocytes, can trigger signalling pathways involved in inflammation and play important roles in a number of cellular processes, such as cell cycle progression, cell survival, proliferation and migration.^50^ S100‐A9 can exist as a homodimer, with its own functions.^51^, ^52^ Meanwhile, we did not observe differential expression of S100‐A8 between the two groups in our proteomic profile results. Our data showed the S100‐A9 concentration was significantly higher in FF‐Exos of PCOS patients compared with normal control, but in supernatant of follicular fluid with no significant difference, which probably suggested that S100‐A9 might exercise its function through exosomes in follicles during PCOS progression. The S100‐A9‐enriched exosomes might be secreted by granulosa cells, ovarian inflammatory cells, peripheral leucocytes or other cell types during PCOS process, affect the crosstalk between granulosa cells and local/ distant environment.

Indeed, we observed that S100‐A9‐enriched exosomes could significantly up‐regulate expression of several pro‐inflammatory factors and chemokines in KGN cell lines, as well as promote NF‐κB pathway activation. Importantly, the specific inhibitor of NF‐κB pathway, BAY‐117082, significantly blocked S100‐A9‐induced inflammation. Our results were consistent with previous study which found S100‐A9 protein in exosomes from chronic lymphocytic leukaemia cells promotes NF‐kB activity.^53^ We also found exosome‐enriched S100‐A9 disturbed steroidogenesis by deregulation of CYP17 mRNA expression via NF‐κB pathway‐dependent manner. Previous studies reported S100‐A9 protein could induce specific cytokine secretion, such as IL‐1, IL‐6 and IL‐8, which in turn enhance the expression of S100‐A9.^51^, ^54^, ^55^, ^56^ So we supposed S100‐A9 and inflammatory cytokines might also form part of a feedback loop in follicles. Our results also confirmed S100‐A9 mRNA expression was up‐regulated after exosomal S100‐A9 treatment (Figure 6B). This positive feedback loop can promote a persistent inflammatory response and cause an adverse effect on reproductive functions or other organ systems.

We also used exosomes from follicular fluid of normal and PCOS patients to treat KGN cells. We found TNF‐a, IL‐1 and IL‐8 mRNA expression level was increased in PCOS‐treated group (Figure S2C). But we did not observe significant activation (a little increase trend) in NF‐κB pathway (Figure S2D), which probably attribute to lower concentration of S100‐A9 in mixed protein cargo of follicular fluid exosome when compared with engineered S100‐A9‐enriched exosome. That's why we chose engineered exosomes to study the functional role of S100‐A9. As too many proteins mixed in the follicular fluid, the inflammation effect might be caused by many proteins and different pathways. Our results found exosomes might be one of the mechanisms of inflammatory process regulation. Future studies about specific exosomal or non‐exosomal proteins and their roles in PCOS are needed to further investigate.

In conclusions, we reported the different proteomic profiles of follicular fluid exosomes among healthy and PCOS patients, some of which are important in reproductive signalling pathways. We found for the first time that S100‐A9 protein in exosomes could activate NF‐κB signalling pathways in granulosa cells, increase the production of inflammatory cytokines and disturb steroidogenesis. Our findings suggest the importance of exosomes as extracellular mediators in the pathophysiology of PCOS. Furthermore, this study contributes to the better understanding of exosomal proteins as potential therapeutic target.

## CONFLICT OF INTEREST

All authors confirm that there are no conflicts of interest.

## AUTHOR CONTRIBUTIONS

Kai Wang and Yazhong Ji contributed to conception and design. Han Li and Julei Yao performed the experiments. Han Li wrote the article. Xinwen Chang analysed and interpreted the data. Xin Huang, Qizhi He and Zhijun Shen collected the clinical samples. Kai Wang and Yazhong Ji gave approval of the submitted and final versions.

## ETHICAL APPROVAL

This study was approved by the Scientific and Ethical Committee of the Shanghai First Maternity and Infant Hospital affiliated to Tongji University. Written informed consent to participate was obtained from all participants.

## Data Availability

The data that support the findings of this study are available from the corresponding author upon reasonable request.
