# Supplementary figures and images for: S100‐A9 protein in exosomes derived from follicular fluid promotes inflammation via activation of NF‐κB pathway in polycystic ovary syndrome
Source: J Cell Mol Med. 2019 Sep 30;24(1):114–25. doi: 10.1111/jcmm.14642 (PMC6933366; doi:10.1111/jcmm.14642)

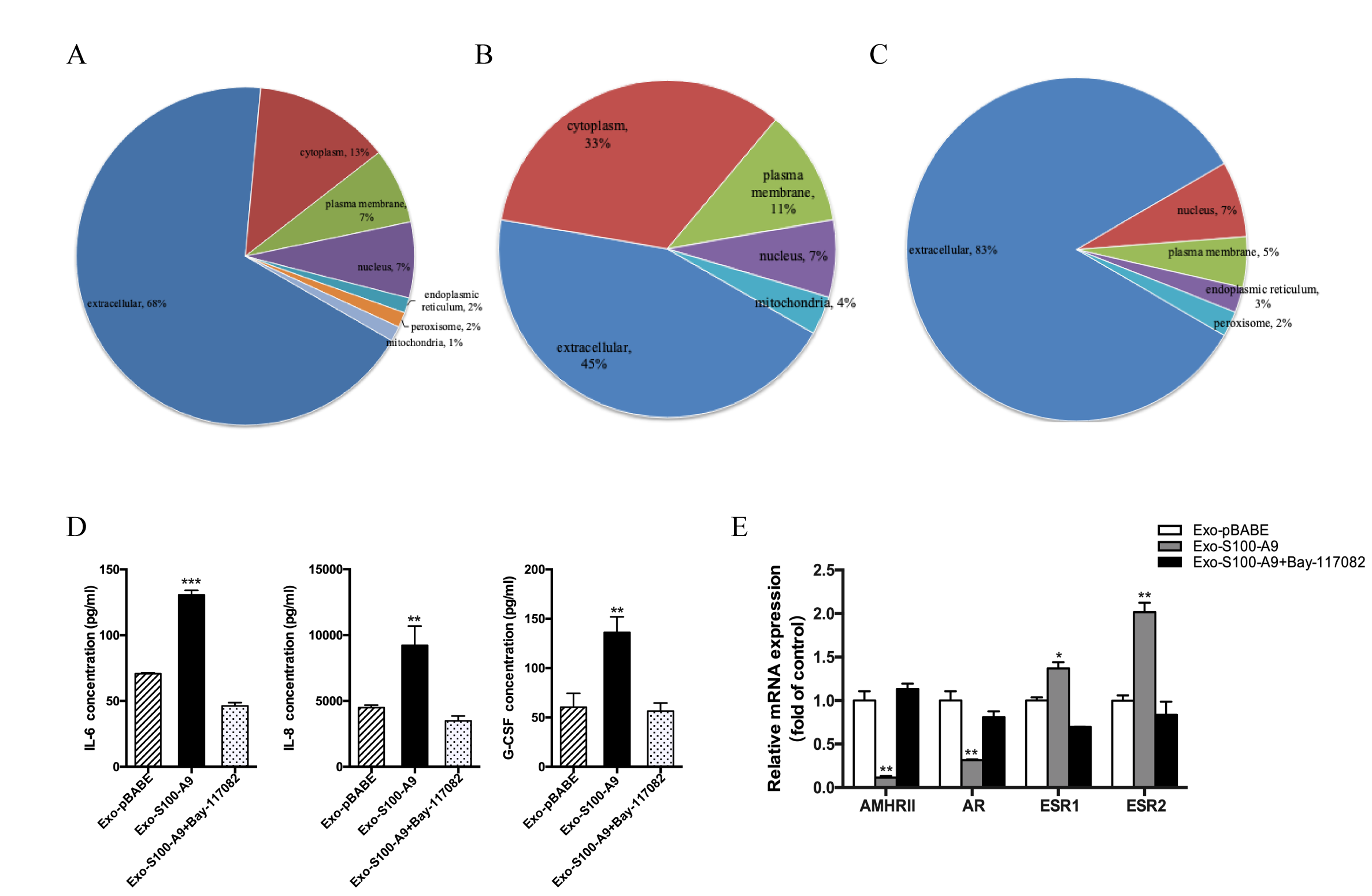

Supplement: Supplementary file 1 [file JCMM-24-114-s001.tif]

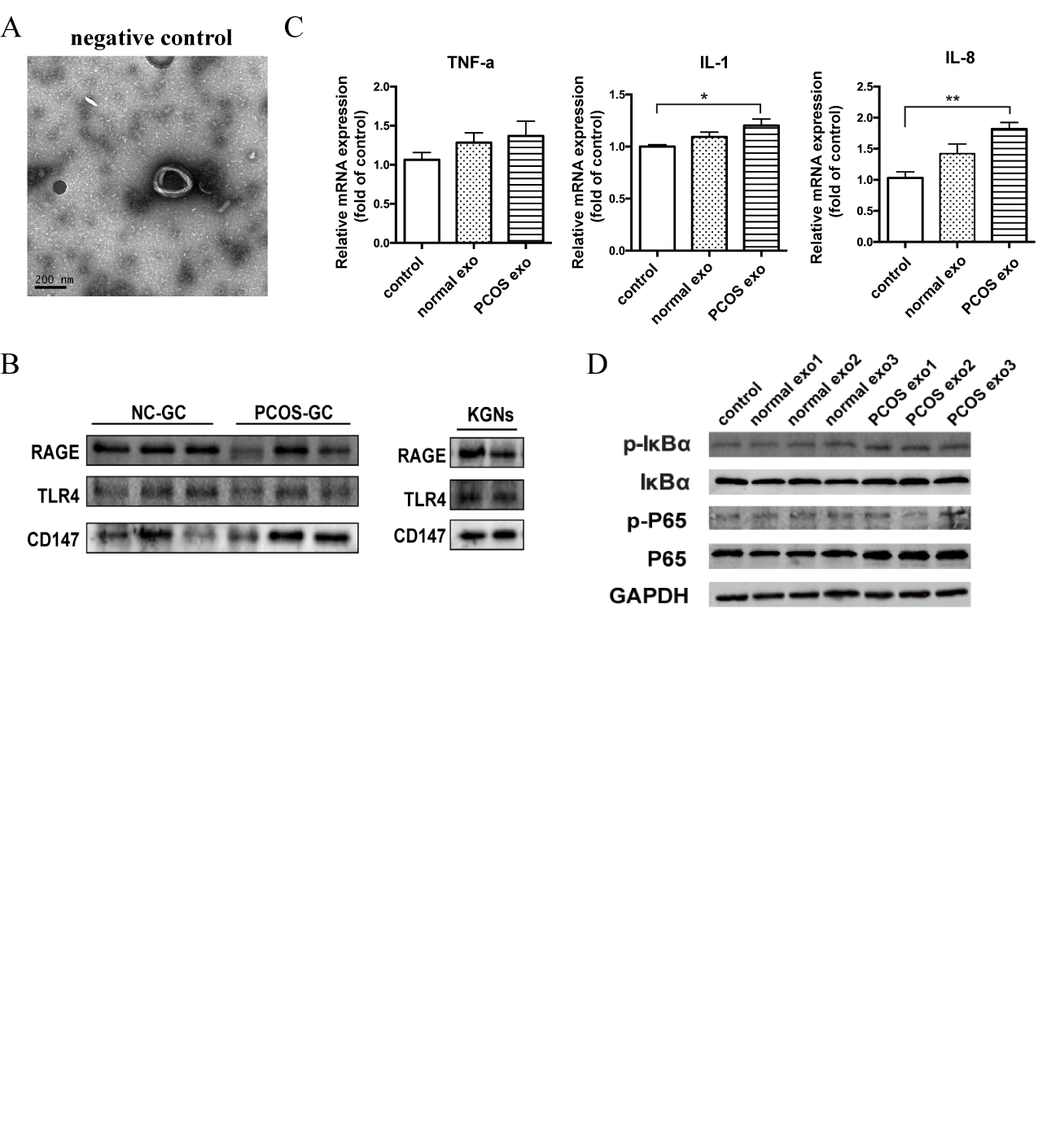

Supplement: Supplementary file 2 [file JCMM-24-114-s002.tif]
