# Supplementary material for: S100‐A9 protein in exosomes derived from follicular fluid promotes inflammation via activation of NF‐κB pathway in polycystic ovary syndrome
Source: J Cell Mol Med. 2019 Sep 30;24(1):114–25. doi: 10.1111/jcmm.14642 (PMC6933366; doi:10.1111/jcmm.14642)
Supplement: Supplementary file 3 [file JCMM-24-114-s003.doc]

**Supplementary table 1. PCR primer sequences**

| **Gene** | **Forward primer (5’-3’)** | **Reverse primer (5’-3’)** |
| --- | --- | --- |
| GAPDH | CGCTGAGTACGTCGTGGAGTC | GCTGATGATCTTGAGGCTGTTGTC |
| TNF-α | CCCTGGTATGAGCCCATCTATC | CAGGGCAATGATCCCAAAGTA |
| IL-1 | GAAATGATGGCTTATTACAGTGGC | GCTGTAGTGGTGGTCGGAGATT |
| IL-6 | AGCCACTCACCTCTTCAGAACG | CAGTGCCTCTTTGCTGCTTTC |
| IL-8 | AGACATACTCCAAACCTTTCCACC | ACAACCCTCTGCACCCAGTT |
| S100A9 5′UTR | CACTCTGTGTGGCTCCTCG | GTTCCAGCTGCGACATTTTG |
| CYP17 | GTAACCGTCTCCTCCTGCTG | ACTTCTGTGCCCTTGTCCAC |
| CYP19A1 | GAGAATTCATGCGAGTCTGGA | CATTATGTGGAACATACTTGAGGACT |
| LHR | CTTGGAGGATGGCTCTTTTCT | CATGGGGAAGCAAATACTGAC |
| AMH | CGC CTG GTG GTC CTA CAC | GAA CCT CAG CGA GGG TGT |
| AMHR-II | TGT GTT TCT CCC AGG TAA TCC | AAT GTG GTC GTG CTG TAG GC |
| ESR1 | CTTGCTCTTGGACAGGAACC | TCCTCTCCCTGCAGATTCAT |
| ESR2 | TGCGGAACCTCAAAAGAGTC | CATCCCTCTTTGAACCTGGA |
| AR | ACCAGCTCACCAAGCTCCT | GCTTCACTGGGTGTGGAAAT |
