# Supplementary material for: S100‐A9 protein in exosomes derived from follicular fluid promotes inflammation via activation of NF‐κB pathway in polycystic ovary syndrome
Source: J Cell Mol Med. 2019 Sep 30;24(1):114–25. doi: 10.1111/jcmm.14642 (PMC6933366; doi:10.1111/jcmm.14642)
Supplement: Supplementary file 4 [file JCMM-24-114-s004.doc]

**Supplementary table 2. Western blot antibodies**

| **Antibody** | **Information** |
| --- | --- |
| anti-S100-A9 | ab92507, 1:1000; Abcam |
| anti-Angiotensinogen | ab108334, 1:1000; Abcam |
| anti-APMAP | ab81176, 1:1000; Abcam |
| anti-Peroxiredoxin 6 | ab133348, 1:1000; Abcam |
| anti-IκB alpha (phospho Ser32/36) | #9246, 1:1000; CST |
| anti-IκB alpha | ab32518, 1:1000; Abcam |
| anti-NF-κB p65 (phospho S536) | ab76302, 1:1000; Abcam |
| anti-NF-κB p65 | ab32536, 1:2000; Abcam |
| exosome-associated protein markers  (CD81, and Hsp70) | EXOAB-KIT-1, 1:1000; SBI |
| Alix | #2171, 1:1000; CST |
| Calnexin | ab92573, 1:20000; Abcam |
| MG130 | Ab52649, 1:1000; Abcam |
| Apolipoprotein A1 | ab52945, 1:1000; Abcam |
| Ablumin | ab207327, 1:2000; Abcam |
| TLR4 | AF1478-SP, 1ug/ml; RD |
| EMMPRIN | 376-020, 1ug/ml; Ancell |
| RAGE | AF1145-SP, 1ug/ml；RD |
| anti-GAPDH | #5174, 1:4000; CST |
| anti-rabbit-IgG | #7074, 1:5000; CST |
| anti-mouse-IgG | #7076, 1:5000; CST |
